# Intelligent Debonding Detection in GFRP Rock Bolts via Piezoelectric Time Reversal and CNN-SVM Model

**DOI:** 10.3390/s25237208

**Published:** 2025-11-26

**Authors:** Zhenyu Zhang, Yang Liu, Yixuan Bai, Jianfeng Si, Zhaolong Zhang, Shengwu Tu

**Affiliations:** 1 State Key Laboratory of Precision Blasting, Jianghan University, Wuhan 430056, China; 2 Hubei Key Laboratory of Blasting Engineering, Jianghan University, Wuhan 430056, China; 3 School of Resources and Environmental Engineering, Wuhan University of Science and Technology, Wuhan 430081, China; 4 Shanghai Jinyi Inspection Technology Co., Ltd., Shanghai 201900, China

**Keywords:** debonding detection, GFRP rock bolts, time reversal, CNN-SVM

## Abstract

To address the challenge of detecting debonding damage in glass-fiber-reinforced polymer (GFRP) rock bolt anchorage structures, this study proposes a time reversal detection method based on piezoelectric sensing and a Convolutional Neural Network–Support Vector Machine (CNN-SVM) model. Through COMSOL 6.1 numerical simulations and laboratory experiments, the influence of debonding length, location, and quantity on the characteristics of detection signals was investigated. The results indicate that an increase in debonding length leads to a rise in the amplitude of the focused signal, a reduction in the main peak frequency, and greater energy concentration around the main peak. Specifically, the amplitude increased by 10.96% (simulations) and 54.9% (experiments) for lengths from 0 to 1200 mm, while the peak frequency decreased by 3.43% (simulations) or increased slightly (experiments). When the debonding location changes, the amplitude remains stable, while the main peak frequency increases by 4.94% in simulations and shifts to higher frequencies experimentally, and the energy exhibits an increasing trend. An increase in the number of debonding points results in decreased amplitude, elevated main peak frequency, and more severe wave packet overlap. Multi-defect configurations reduced the amplitude by 16.68% (simulations) and 3% (experiments), with peak frequency increases of up to 3.35%. Based on these characteristics, a CNN-SVM evaluation model was constructed, using the wavelet time–frequency maps of experimental signals as input and the debonding state as output. The model achieved evaluation accuracy rates of 99%, 100%, and 100% under varying debonding lengths from 10 to 100 mm, different debonding positions, and increasing numbers of debonding defects, all exceeding 95%, thereby validating the reliability and high precision of the proposed method.

## 1. Introduction

Glass-fiber-reinforced polymer (GFRP) rock bolts exhibit notable advantages in tunnel engineering and slope stabilization owing to their superior properties, including their light weight, high tensile strength, and corrosion resistance [1,2,3,4,5]. However, under complex and severe engineering conditions, the anchorage structures of GFRP rock bolts are prone to long-term degradation from coupled factors, often resulting in two typical failure modes: rock bolt body cracking and interfacial debonding [6,7]. Environmental factors, such as temperature, humidity, and coupled loads, contribute to long-term degradation of GFRP rock bolts, accelerating anchorage failure mechanisms [8,9]. Some studies confirm that synergistic effects of temperature fluctuations, moisture ingress, and sustained loads significantly degrade GFRP’s interfacial adhesion and tensile strength [10,11]. For instance, accelerated aging tests demonstrate significant degradation in bond strength following cyclic hygrothermal exposure, while coupled thermo–mechanical loads lead to matrix microcracking and fiber–matrix debonding [12]. Rock bolt body cracks directly compromise the load-bearing capacity of the rock bolt [13]. Interfacial debonding manifests in two forms: separation between the rock bolt and grout, and detachment between the grout and surrounding rock mass [14,15]. Collectively, these defects severely degrade the anchoring system’s integrity, potentially triggering catastrophic engineering failures like slope collapse or tunnel lining rupture [16]. Thus, developing advanced detection technologies for GFRP rock bolt anchorage quality and establishing a robust defect evaluation framework are critical to ensuring the long-term stability of geotechnical structures [17,18].

Rock bolt testing technologies are primarily categorized into destructive and non-destructive methods. Destructive testing, exemplified by pull-out tests, can directly quantify anchoring force but irreversibly damages the rock bolt structure [19]. Moreover, techniques like core extraction suffer from high costs and low efficiency [20]. Among non-destructive techniques, fiber Bragg gratings (FBGs) provide high-precision monitoring but necessitate pre-embedded sensors and intricate installation [21]. Ultrasonic-guided waves exhibit insufficient sensitivity for deep defects in composites [22], while strain gauges are confined to single-point measurements, hindering full cross-sectional assessment [23]. Piezoelectric-material-based stress wave detection offers high sensitivity and efficiency, with lead zirconate titanate plates combining actuation and sensing capabilities [24]. However, current methods grapple with challenges like low signal-to-noise ratios and inadequate minor defect identification under complex conditions [25,26]. For instance, guided waves effectively detect surface defects in GFRP rock bolts but lack precision for deep interfacial debonding [27]. Similarly, Hilbert–Huang transform (HHT)-based methods enhance signal-to-noise ratios yet remain inadequate for multifactor-influenced defect characterization [28]. Furthermore, identifying finer-scale damage modes (e.g., fiber breakage, resin cracking) remains challenging for conventional techniques. Specifically, existing non-destructive methods struggle to resolve finer-scale damage modes such as interfacial micro-voids, fiber breakage, or resin cracking due to insufficient sensitivity to localized stiffness loss or signal-to-noise constraints [29]. Thus, developing novel methods integrating multi-physical field sensing and intelligent signal processing is imperative [30].

Time reversal, a signal processing technique based on temporal inversion and re-emission of time-domain signals, demonstrates remarkable advantages in structural health monitoring (SHM) by leveraging its inherent spatiotemporal focusing properties [31]. In engineering practice, time reversal methods have successfully identified corrosion damage in reinforced concrete structures and enabled quality monitoring of prestressed pipe grouting [32]. For composite materials, this technology resolves delamination defect localization in carbon-fiber-reinforced polymers (CFRP) via Lamb wave time reversal with weighted distribution imaging [33]. For rock bolt preload monitoring, piezoelectric sensors employing time reversal principles correlate focused signal amplitude logarithmically with preload. In pipeline inspections, it enhances sensitivity to minor defects [34]. The core mechanism involves noise suppression via time reversal, amplifying subtle defect features through signal reconstruction, and integrating amplitude, energy, and other metrics for quantitative damage evaluation. Studies confirm the method’s unique adaptability to multifactor-coupled complex media, rendering it ideal for GFRP rock bolt quality testing by overcoming traditional limitations, such as a low signal-to-noise ratio (SNR) and insensitivity to minor defects in debonding and crack detection [35].

The rapid advancement of artificial intelligence (AI), especially deep learning, has introduced novel technical approaches for structural damage identification. Convolutional neural networks (CNNs) exhibit exceptional performance in image recognition and fault diagnosis owing to their robust feature extraction capabilities [36]. Researchers have investigated diverse intelligent algorithms for non-destructive testing, including highly fault-tolerant detection methods using feature vector inputs, least-squares support vector machine (LS-SVM) classifiers, and mapping neural networks. These studies demonstrate that integrating advanced feature extraction techniques with classification algorithms can substantially enhance defect identification accuracy [37]. The CNN-SVM model combines CNN’s feature extraction capability with SVM’s robust small-sample classification, enabling effective time–frequency analysis of wavelet-transformed 2D representations for precise GFRP rock bolt debonding detection [38].

To meet the quality inspection demands of GFRP rock bolts, this study introduces an intelligent detection approach integrating piezoelectric sensing and time reversal techniques. The key innovation lies in the fusion of two advanced technologies: (i) piezoelectric time reversal, which exploits spatiotemporal focusing to amplify subtle defect-induced signal distortions in noisy environments; and (ii) a CNN-SVM hybrid model that autonomously extracts discriminative features from time–frequency representations for high-accuracy classification of debonding configurations. This integrated methodology overcomes critical limitations of existing techniques—such as low signal-to-noise ratios (SNR), insensitivity to minor defects, and an inability to quantify multifactor-coupled damage—enabling robust, high-resolution quantification of interfacial debonding defects under complex conditions.

Section 2 elaborates on the experimental design and theoretical foundations of the time reversal method and CNN-SVM model construction; Section 3 verifies the method’s efficacy via numerical simulation experiments; Section 4 conducts a performance analysis of the detection method using laboratory test data; Section 5 implements the CNN-SVM model for damage classification and identification; and Section 6 concludes with a summary of the research findings. While this study focuses on isolated variables (debonding length, location, and quantity) to elucidate fundamental signal–response mechanisms, real-world degradation often involves coupled factors (e.g., simultaneous mechanical loads and environmental effects). Future work will address these interactions.

## 2. Methodology and Theoretical Framework

### 2.1. Time Reversal Method

The time reversal method exploits Lamb wave reciprocity in elastic media, enabling damage detection/localization through spatiotemporal reconstruction of wave propagation paths via temporal inversion and re-emission [39].

**Figure 1:**
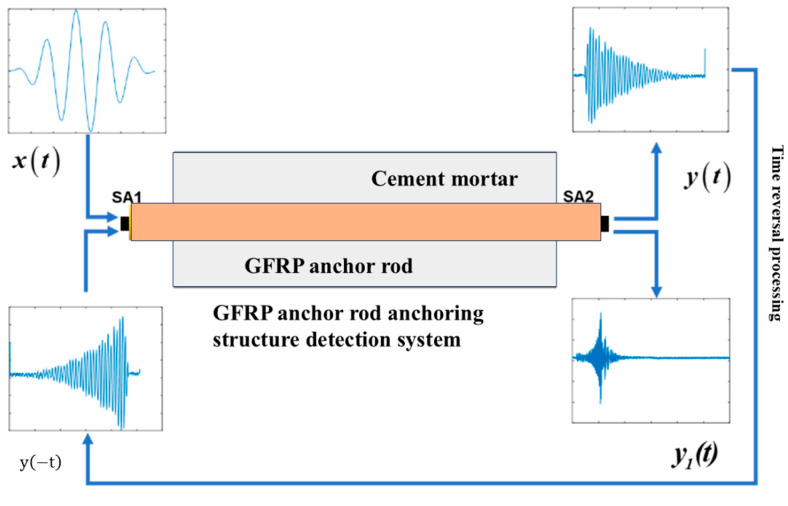
Schematic of time-reversal-based piezoelectric sensing for GFRP rock bolt debonding detection.

As illustrated in Figure 1, the key steps of the time reversal method can be summarized as follows: An incident voltage signal $V_{A}\left(\omega \right)$ with center frequency ω excites the piezoelectric transducer (SA1), where it undergoes conversion to mechanical strain via the electromechanical coupling coefficient $k_{A}\left(\omega \right)$. The resultant strain wave subsequently propagates to the receiving piezoelectric transducer (SA2). For any given piezoelectric sensor, the product of electromechanical coupling coefficient $k_{A}\left(\omega \right)$ and mechanoelectrical coupling coefficient $g_{A}\left(\omega \right)$ exhibits proportionality to the piezoelectric material’s dielectric constant $\varepsilon_{PZT}$, maintaining a constant value [40,41].

(1) $$\varepsilon_{A}\left(\omega \right)=k_{A}\left(\omega \right)V_{A}\left(\omega \right)$$

(2) $$k_{A}\left(\omega \right)\times g_{A}\left(\omega \right)\propto \frac{1}{\varepsilon_{PZT}}$$

For the cylindrical GFRP bolt (diameter d = 16 mm) excited at 75 kHz, the frequency–thickness product f·d = 1200 Hz corresponds to the dominant fundamental antisymmetric Lamb wave mode ($A_{0}$) in plate-like structures. The $A_{0}$ mode exhibits strong sensitivity to interfacial stiffness changes due to its out-of-plane particle motion, enabling effective debonding detection.

The structural transfer function G(ω) transforms the strain from the excitation point to the reception point, whereupon the transmitted strain is detected by piezoelectric transducer SA2 and reconverted to an electrical signal, expressed as

(3) $$V_{B}\left(\omega \right)=g_{B}\left(\omega \right)G\left(\omega \right)k_{A}\left(\omega \right)V_{A}\left(\omega \right)$$

Here, $g_{B}\left(\omega \right)$ denotes the mechanoelectrical coupling coefficient of piezoelectric transducer SA2. Through Fourier transform analysis, the time-reversed signal’s frequency-domain representation exhibits complex conjugate symmetry, expressed as $V_{B}^{*}\left(\omega \right)$. Consequently, the time-reversed signal received by SA1 (processed by SA2) is formulated as

(4) $$\dot{V_{A}}\left(\omega \right)=g_{A}\left(\omega \right)G\left(\omega \right)k_{B}\left(\omega \right)\dot{V_{B}^{*}}\left(\omega \right)$$

By substituting Equation (3) into Equation (4), the frequency-domain signal reconstruction can be expressed as

(5) $$V_{A}\left(\omega \right)=g_{A}\left(\omega \right)G\left(\omega \right)k_{B}\left(\omega \right)g_{B}^{*}\left(\omega \right)G^{*}\left(\omega \right)k_{A}^{*}\left(\omega \right)V_{A}^{*}\left(\omega \right)$$

For piezoelectric transducer units from the same production batch and model, their parameters can be assumed to exhibit consistency and stability. Consequently, frequency-domain signal reconstruction can be simplified as:

(6) $$V_{A}\left(\omega \right)=CG\left(\omega \right)G^{*}\left(\omega \right)V_{A}^{*}\left(\omega \right)$$

In Equation (6), *C* represents a constant determined by the piezoelectric transducer’s (PZT) dielectric constant $\varepsilon_{PZT}$.

(7) $$V_{A}^{\prime }\left(t\right)=\frac{1}{2\pi }\int_{-\infty }^{\infty }G\left(\omega \right)G^{*}\left(\omega \right)V_{A}^{*}\left(\omega \right)e^{j\omega t}d\omega$$

When the transmission path remains undamaged, the structural transfer function G(ω) maintains its center frequency without dispersion, exhibiting purely scalar wave propagation properties. Consequently, the reconstructed time-domain signal shows proportional scaling to the original signal with preserved waveform morphology [42]. Conversely, transmission path damage induces waveform distortion in the reconstructed signal, enabling quantitative damage assessment through reconstructed original signal differential analysis [43].

### 2.2. CNN-SVM Model

**Figure 2:**
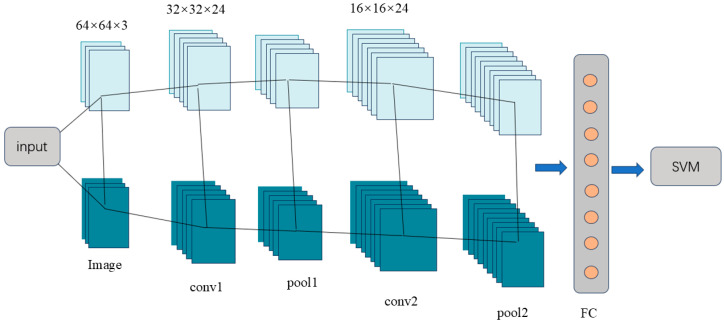
Architecture of the CNN-SVM classification model.

To address the limited generalization capability of traditional CNN models, this study proposes a CNN-SVM hybrid architecture. The model employs CNN’s convolutional pooling hierarchical structure to extract spatiotemporal features, while replacing the Softmax classifier with a support vector machine (SVM). An SVM performs sample classification by constructing an optimal hyperplane, as illustrated in Figure 2. This architecture integrates CNN’s superior feature extraction with the SVM’s small-sample classification performance [44].

(1) Convolution layer and activation layer [45,46]

Convolutional layers extract image features via learnable filters, with performance scaling with layer depth. The operation is defined as

(8) $$R\left(x,y\right)=\sum_{i=1}^{k}\sum_{j=1}^{p}M\left(i,j\right)*A\left(x+1,y+j\right)$$

where A is the input feature matrix,M is the kernel, and $\left(k,p\right)$ are the filter dimensions. Activation layers employ nonlinear functions (e.g., ReLU) for computational efficiency and gradient stability. ReLU’s piecewise linearity enhances feature discrimination and mitigates vanishing gradients, improving model robustness.

(2) Pooling layer [47]

Pooling layers downsample feature maps via max/average operations, reducing spatial dimensions while preserving critical features. This enhances computational efficiency and translational invariance. The max pooling operation is defined as

(9) $$y_{ij}^{k}=\max\left(x_{i^{\prime }j^{\prime }}^{k}:i\leq i^{\prime }<i+h,\;j\leq j^{\prime }<j+q\right)$$

where (h×q) is the pooling window size.

(3) Flattening and fully connected (FC) layers [48,49]

Flattening layers reshape convolutional outputs into 1D vectors for FC processing. FC layers integrate global features, enhancing model capacity via dense connections.

(4) Support vector machines (SVMs) [50]

SVMs optimize classification via structural risk minimization, constructing a maximum-margin hyperplane:

(10) $$f\left(x\right)=w^{T}\phi \left(x\right)+b$$

Kernel tricks enable nonlinear separability. Multi-class extensions and parameter optimization further improve accuracy, particularly in high-dimensional, small-sample scenarios [51,52].

### 2.3. Experimental Design

**Figure 3:**
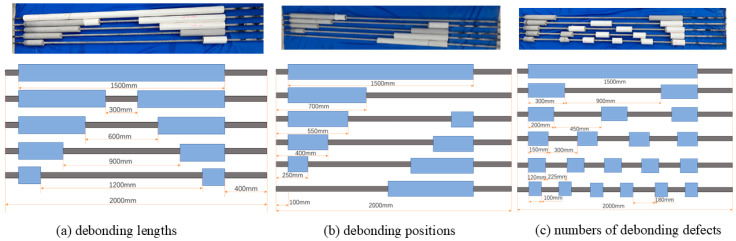
Design of GFRP rock bolt specimens with controlled debonding configurations.

This study addresses typical defect-inducing factors in grouting operations, including insufficient grout filling, gravitational segregation, borehole wall collapse, inappropriate water–cement ratios, and improper grout injection techniques, all of which contribute to interfacial debonding defects [53,54,55]. As illustrated in Figure 3, three specimen groups with distinct debonding configurations were designed based on standard engineering practices for GFRP rock bolts in tunnel support and slope stabilization, where typical field anchorage lengths range from 1.2 to 2.0 m. The laboratory anchorage length of 1500 mm was validated through similarity principles to replicate field conditions, with a total specimen length of 2000 mm and asymmetric free ends (100 mm left, 400 mm right) simulating stress concentration zones in actual rock masses. For defect simulation, debonding was artificially induced by precisely excising sections of the PVC mold during specimen fabrication, creating air-filled voids at the GFRP–mortar interface. This directly replicates the interfacial separation (debonding) observed in grouting defects, such as incomplete filling and segregation. Defect dimensions (length, location, count) were systematically controlled to reflect practical failure scenarios: Group 1 employed 300–1200 mm defect lengths, covering critical damage thresholds; Group 2 varied defect locations to assess boundary effects; and Group 3 used multi-defect configurations, mimicking distributed delamination.

## 3. Finite Element Modeling and Numerical Analysis

### 3.1. Multiphysics Model Setup and Material Properties

**Figure 4:**
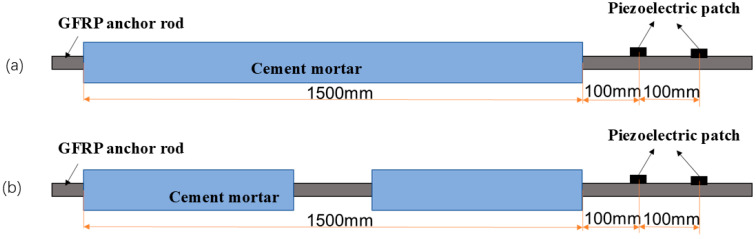
GFRP rock bolt structure debonding detection model diagram: (**a**) without debonding defect; (**b**) with debonding defect.

Utilizing the structural mechanics module in COMSOL Multiphysics, a 3D axisymmetric model was developed to simulate the cylindrical GFRP rock bolt–cement mortar composite system. Figure 4 depicts the numerical model, consisting of a 16 mm diameter GFRP rock bolt with a total length of 2000 mm. The rock bolt was concentrically embedded within a 1500 mm long cement mortar annulus, featuring outer and inner diameters of 40 mm and 16 mm, respectively. Simulations excited the $A_{0}$ Lamb mode at 75 kHz, which was selected for its optimal sensitivity to interfacial debonding in the low-frequency-thickness regime.

A fully coupled multiphysics constraint was applied to the interface between the inner surface of the mortar and the outer surface of the GFRP bolt to ensure mechanical continuity. The GFRP rock bolt was modeled as an orthotropic linear elastic material, with direction-dependent properties defined by Young’s modulus, Poisson’s ratios, shear moduli, and density matrices. Piezoelectric components employed the built-in PZT-4 material with a stress–charge constitutive formulation, parameterized by the elasticity matrix, piezoelectric coupling matrix, and relative permittivity. Cement mortar was treated as an isotropic linear elastic material, characterized by Young’s modulus, Poisson’s ratio, and density. Table 1 summarizes the complete mechanical property datasets for all materials.

### 3.2. Boundary Conditions and Mesh Sensitivity Analysis

To address the interface settings in our finite element modeling, fully coupled multiphysics constraints were rigorously applied at all critical interfaces to ensure mechanical–electrical continuity. Specifically, for the mortar–GFRP bolt interface, a bidirectional mechanical coupling was enforced to simulate perfect bonding under undamaged conditions, guaranteeing strain continuity. The fully coupled multiphysics constraints were implemented at all material interfaces, including the cement mortar inner surface, GFRP rock bolt outer surface, PZT plates, and air voids within defects, to ensure mechanical–electrical continuity. For defects such as air gaps within debonding zones, zero-charge boundaries were applied to replicate stress wave scattering, and explicit mechanical discontinuities were defined to disrupt strain transfer. PZT-4 piezoelectric plates were actuated through circuit excitation, facilitating energy conversion from electrical to mechanical domains via the constitutive stress–charge formulation. Stress wave propagation was numerically simulated by imposing zero-charge and symmetry boundaries along the X and Y directions, while enforcing charge conservation along the rock bolt axis in the Z direction using COMSOL’s electrostatics–piezoelectric interface. At the PZT–bolt interface, impedance-matched low-reflection boundaries were implemented to minimize spurious wave reflection. Ground boundaries with zero potential were applied at both ends, and impedance-matched low-reflection boundaries were implemented at PZT–bolt interfaces to minimize wave reflections. Boundary probes positioned at PZT–anchor interfaces monitored wave propagation through real-time voltage signals sampled at 1 MHz, which was sufficient to resolve the 75 kHz Hanning-windowed excitations.

A hybrid tetrahedral meshing strategy combining automatic and manual techniques was employed with size-adaptive partitioning. The mesh parameters were optimized for each component: GFRP rock bolt elements ranged from 0.121 to 12.1 mm, mortar elements from 0.909 to 21.2 mm, and PZT plates maintained a maximum element size of 1.25 mm. Experimental calibration determined the optimal excitation as a 3-cycle, 75 kHz Hanning-windowed sine wave, providing balanced performance while maintaining strong noise immunity.

### 3.3. Parametric Study of Debonding Effects on Wave Propagation

(1) Effect of debonding defect length on focused signal

**Figure 5:**
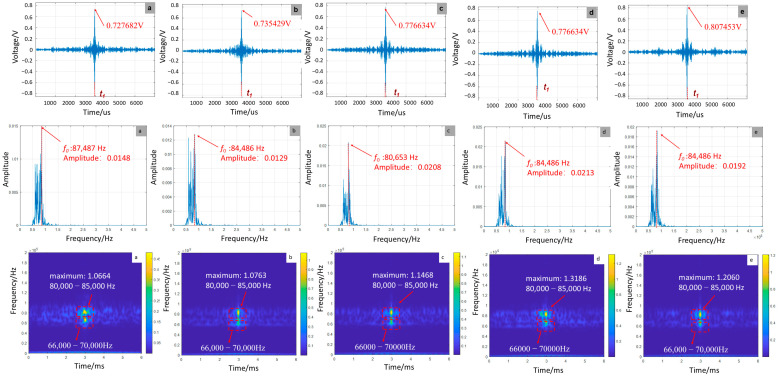
Time-domain, frequency-domain, and time-frequency-domain responses for varying debonding lengths: (**a**) no defect; (**b**) 300 mm; (**c**) 600 mm; (**d**) 900 mm; (**e**) 1200 mm (simulation).

As shown in Figure 5, numerical simulations demonstrate a significant dependence of focused signal characteristics on debonding defect length. As the debonding length increases from 0 to 1200 mm, the signal amplitude rises from 0.727682 V to 0.807453 V, reflecting a 10.96% increase attributable to reduced structural stiffness in the GFRP rock bolt system. Frequency-domain analysis reveals a concomitant downward shift in the main peak frequency from 87,487 Hz to 84,486 Hz. Time–frequency analysis further shows enhanced energy concentration within the 80,000 Hz–85,000 Hz range, with peak energy values increasing from 1.0664 to 1.3186, illustrating the pronounced modulation effect of stiffness reduction on wave energy distribution.

(2) Effect of delamination defect location on focused signal

**Figure 6:**
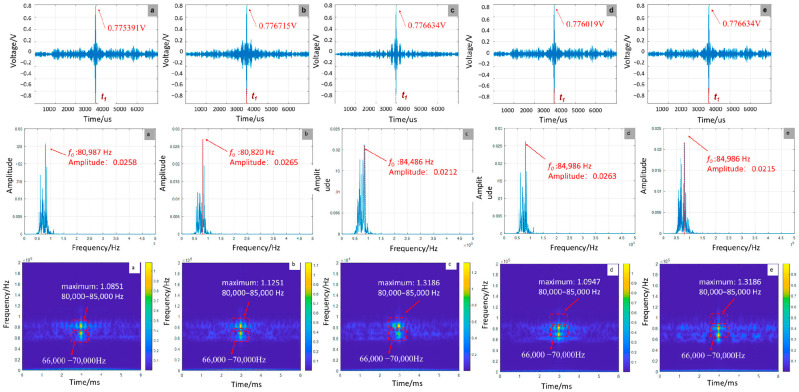
Time-domain, frequency-domain, and time-frequency-domain responses for varying debonding locations: (**a**) 0 mm; (**b**) 150 mm; (**c**) 300 mm; (**d**) 450 mm; (**e**) 600 mm (simulation).

As shown in Figure 6, the debonding defect location exhibits negligible influence on signal amplitude, with variations limited to ±0.15% around a mean value of 0.7762022 V, demonstrating amplitude invariance to positional changes. In contrast, frequency-domain characteristics show pronounced location dependence. As the defect moves from the left to the right end, the peak frequency increases from 80,987 Hz to 84,986 Hz. Time–frequency analysis reveals distinct energy distribution patterns: central defects induce energy dispersion in the 66,000 Hz–70,000 Hz range, while boundary-proximal defects enhance energy concentration. This behavior stems from wave interference between boundary reflections and defect-scattered waves.

(3) Effect of the number of debonding defects on the focus signal

**Figure 7:**
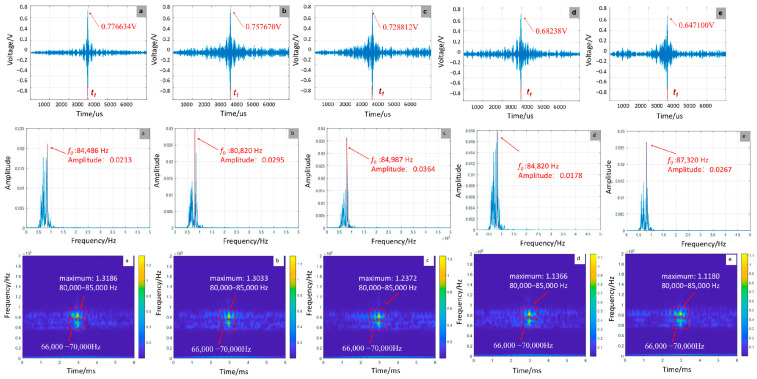
Time-domain, frequency-domain, and time-frequency-domain responses for varying numbers of debonding defects: (**a**) 1; (**b**) 2; (**c**) 3; (**d**) 4; (**e**) 5 (simulation).

As shown in Figure 7, the amplitude of the focal signal demonstrates a monotonic decrease with increasing debonding defects, declining from 0.776634 V for a single defect to 0.647100 V for five defects, representing a 16.68% reduction. This attenuation arises from energy dissipation caused by repeated interfacial wave reflections. Frequency-domain analysis indicates a progressive shift of the main peak frequency toward higher frequencies, increasing from 84,486 Hz to 87,320 Hz as defect count rises, which results from the superposition of higher-order harmonics generated by multiple defects. Time–frequency analysis corroborates this trend, showing a decrease in main peak energy from 1.3186 to 1.1180, accompanied by energy redistribution toward the 80,000 Hz–85,000 Hz band. These observations underscore the modulation of wave propagation paths through multi-defect interactions.

**Figure 8:**
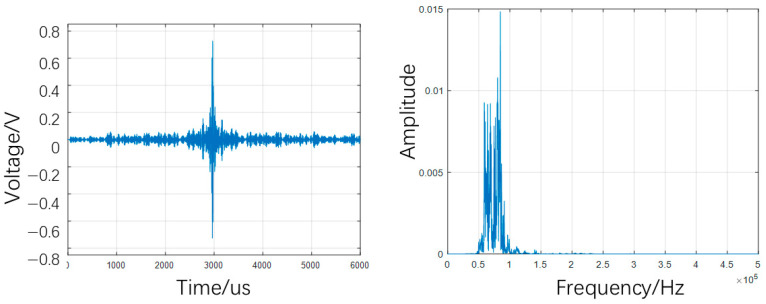
Typical time-domain and frequency-domain plots for no-defect case.

**Figure 9:**
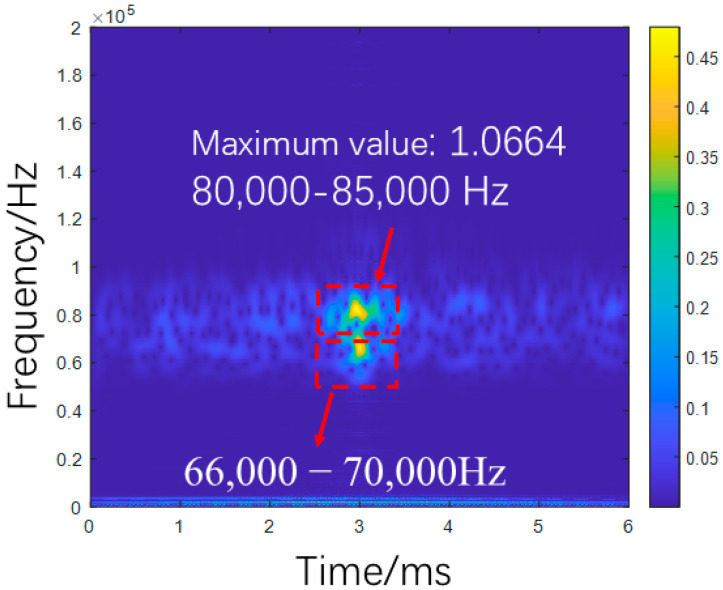
Typical time–frequency plot for no-defect case.

The typical time-domain, frequency-domain, and time–frequency plots for the defect-free case are presented in Figure 8 and Figure 9.

## 4. Experimental Validation and Signal Characterization

### 4.1. Fabrication of GFRP Anchorage Specimens with Controlled Defects

The specimens were fabricated using 16 mm diameter, 2 m long GFRP rock bolts, conforming to similarity testing principles. The anchorage structure utilized a concrete mix with a cement/fine, sand/water mass ratio of 5:3:2, prepared with ordinary cement (grade 32.5). During casting, each GFRP rock bolt was axially aligned within a 50 mm inner diameter PVC pipe (length: 1700 mm), and concrete was poured into the annular space. The specimens underwent 28-day standard laboratory curing. Debonding and slip defects were artificially introduced to simulate field conditions. The PVC pipe was excised using precision cutting tools, and specimens were trimmed to meet dimensional tolerances. For optimal sensor adhesion, the GFRP surface was polished at 100 mm and 200 mm from the anchorage end using an angle grinder. The final specimen configuration is depicted in Figure 3.

### 4.2. Piezoelectric Sensing System and Data Acquisition Protocol

**Figure 10:**
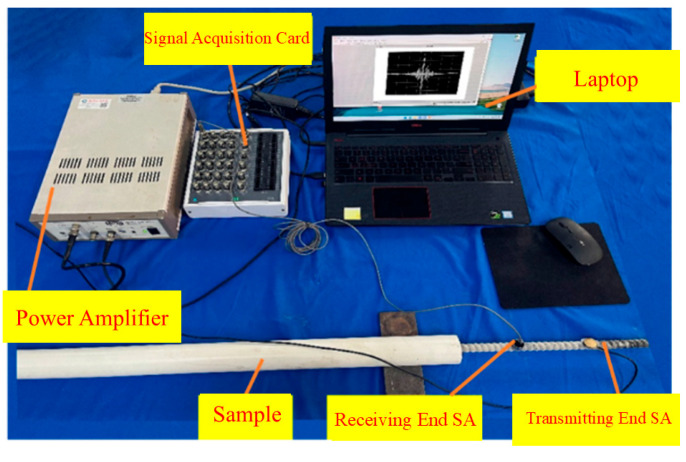
Time-domain signals and frequency spectra for varying debonding lengths (0–1200 mm).

Figure 10 presents the experimental setup for assessing glass-fiber-reinforced polymer (GFRP) rock bolts with interfacial debonding defects. The system integrates five core components:a data acquisition device, a laptop, a power amplifier, test specimens, and smart aggregates (SAs). The NI-usb 6363 (National Instruments, Austin, TX, USA) serves as the central hub for signal generation and acquisition, coupled with a TREK 603 (Trek Inc., Lockport, NY, USA) power amplifier delivering 50× amplification. Two SAs function as transmitter (SA1) and receiver (SA2). A Hann-window-modulated, five-cycle sine wave (75 kHz center frequency) was selected as the excitation signal to reduce spectral leakage. The signal, generated by the piezoelectric system’s arbitrary waveform generator, operated in single-external-trigger mode with the following parameters: 1024 data points, 1 μs interval, unity gain, and 1024 μs total duration. Signal acquisition utilized a ±0.08 V range, 2 MHz sampling frequency, 8192-point resolution, and 4.096 ms acquisition window. SA1 transmitted the signal through the GFRP anchorage system, and SA2 captured the modified waveform after it interacted with the structure. Time-reversal processing was applied to the received signal prior to retransmission, leveraging multipath coherence to enhance energy focusing at SA2. Each test was repeated 20 times, with arithmetic averaging ensuring statistical robustness in data analysis.

### 4.3. Time–Frequency Signal Response to Debonding Variants

(1) Effect of debonding defect length on signal characteristics

**Figure 11:**
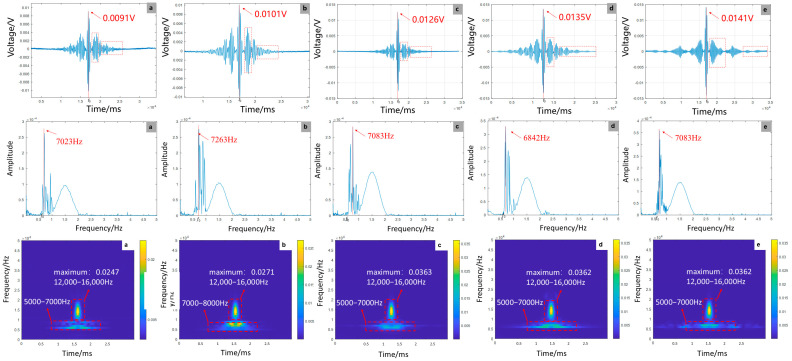
Time-domain, frequency-domain, and time-frequency-domain responses for varying debonding lengths: (**a**) no defect; (**b**) 300 mm; (**c**) 600 mm; (**d**) 900 mm; (**e**) 1200 mm (experiment).

As shown in Figure 11, experimental results reveal a significant positive correlation between debonding defect length and focused signal amplitude, with a 54.9% amplitude enhancement observed. This phenomenon accompanies a peak frequency shift from 7023 Hz to 7083 Hz and a 13.1% increase in energy concentration. While numerical simulations predict a more modest 10.96% amplitude growth, they exhibit divergent frequency trends, showing reduction versus experimental increases. This discrepancy likely stems from material heterogeneity and complex boundary effects in experiments, which contrast with the idealized homogeneous conditions assumed in simulations. Time–frequency analysis demonstrates consistent energy concentration patterns between experimental and simulated data, validating the theoretical framework that debonding reduces structural stiffness. However, experimental measurements display pronounced signal fluctuations, reflecting real-world interfacial friction and microcrack dynamics. The findings highlight that extended defects substantially degrade dynamic stiffness, emphasizing the utility of frequency-domain monitoring—particularly peak frequency shifts—for early debonding detection in GFRP rock bolt systems.

(2) Effect of delamination defect location on signal characteristics

**Figure 12:**
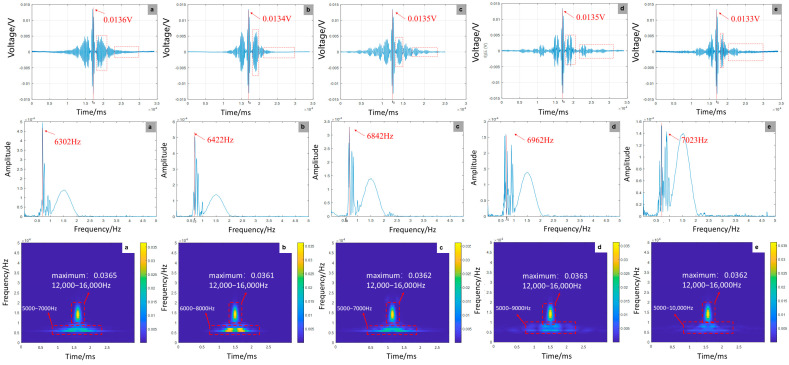
Time-domain, frequency-domain, and time-frequency-domain responses for varying debonding locations: (**a**) 0 mm; (**b**) 150 mm; (**c**) 300 mm; (**d**) 450 mm; (**e**) 600 mm (experiment).

As shown in Figure 12, experimental results indicate that defect location has minimal impact on signal amplitude (±1.19% variation) but significantly influences waveform clarity. The wave packet achieves optimal clarity at the mid-span position (300 mm), whereas boundary locations (0/600 mm) exhibit severe spectral aliasing. This finding aligns with numerical simulations showing a frequency-domain peak shift from 80,987 Hz to 84,986 Hz. While simulations captured frequency modulation, experiments revealed critical aliasing thresholds at boundaries, underscoring the spatial dependence of wave interference. Time–frequency analysis demonstrates stronger boundary reflections in experiments, attributed to higher constraint stiffness at anchoring ends compared to idealized simulation conditions. The interference between boundary reflections and defect scattering presents greater complexity in experimental data, requiring quantification through wave equation solutions and interface impedance modeling to address multimodal coupling effects.

(3) Effect of the number of debonding defects on signal characteristics

**Figure 13:**
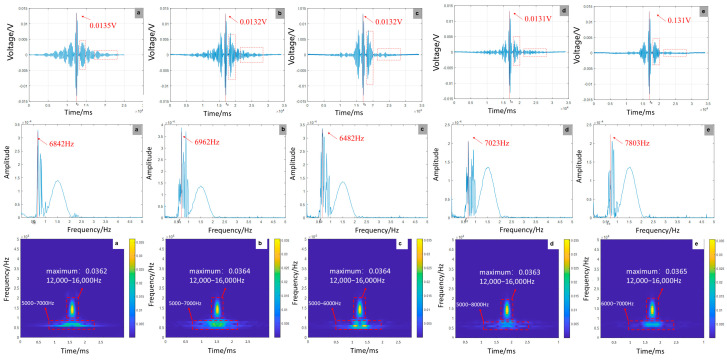
Time-domain, frequency-domain, and time-frequency-domain responses for varying numbers of debonding defects: (**a**) 1; (**b**) 2; (**c**) 3; (**d**) 4; (**e**) 5 (experiment).

As shown in Figure 13, experimental and numerical results consistently show that multi-defect configurations lead to signal amplitude attenuation, with experimental and simulated reductions measuring 3.0% and 16.68%, respectively. These configurations also induce high-frequency shifts, reaching 7803 Hz in experiments compared to 87,320 Hz in simulations. The experimental amplitude decay follows a more gradual trend, likely due to energy dissipation at material interfaces and inherent damping effects not fully represented in simulations. Time–frequency analysis reveals aligned energy migration trends between experiments and simulations, though experimental energy attenuation rates are significantly higher (11.2% vs. 15.2%). This discrepancy supports the multi-interface reflection theory while underscoring simulation limitations in capturing dissipative losses. Numerical simulations systematically overestimate defect-induced amplitude reduction, highlighting the need for damping correction factors in engineering predictions. These findings align with established practices for high-damping systems, where simulation–empirical discrepancies often exceed ±20% tolerance thresholds.

## 5. Intelligent Defect Identification via CNN-SVM

### 5.1. Model Parameter Settings and Preprocessing

This study developed a CNN-SVM hybrid algorithm using MATLAB R2023b, with time–frequency images serving as model inputs. The network architecture consists of sequential convolutional layers, batch normalization layers, ReLU activation functions, and pooling layers, featuring two iterative processing stages before the final fully connected layer. The CNN model was trained using the Adam optimizer with an initial learning rate of 0.001 and a decay factor of 0.01 over 100 epochs, incorporating dynamic learning rate adjustment during training. Following CNN feature extraction, the outputs were classified using an SVM with grid-optimized hyperparameters. Complete architectural specifications are provided in Table 2.

### 5.2. Analysis of Classification Results

**Figure 14:**
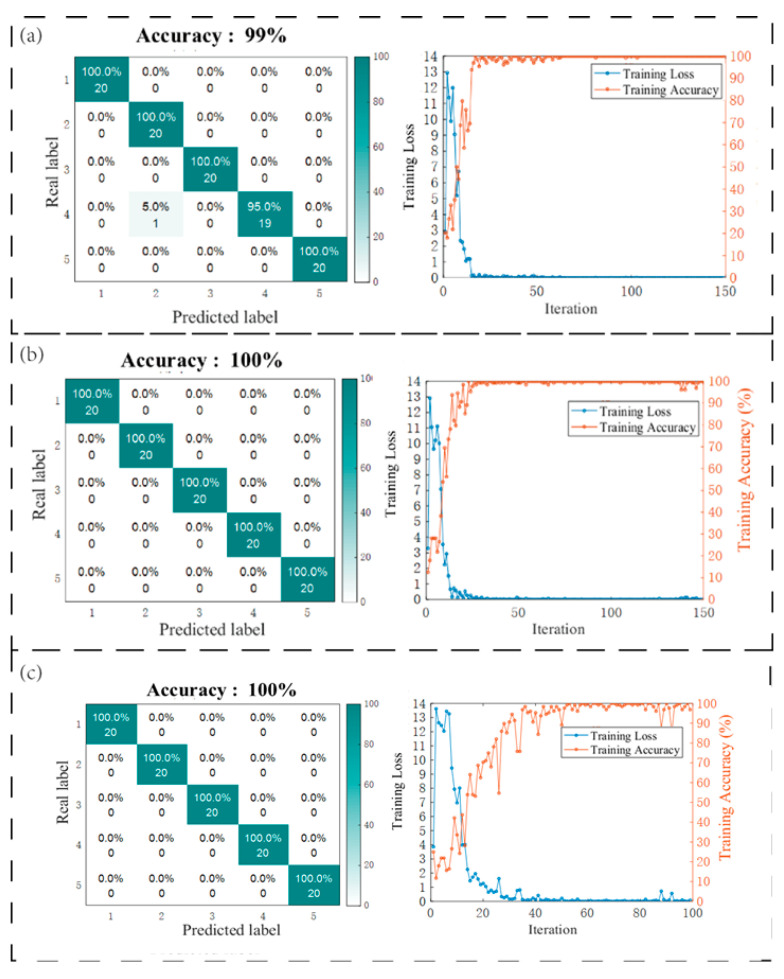
Model prediction results: (**a**) debonding lengths; (**b**) debonding positions; (**c**) number of debonding defects.

This experimental study systematically evaluated three critical parameters of delamination defects—length, location, and quantity—using a CNN-SVM hybrid architecture for feature extraction and classification. For each anchor rod under different working conditions, 100 signal samples were independently collected. The training set (80% of samples) and test set (20% of samples) were rigorously partitioned with no overlap. As shown in Figure 14, the analysis of defect length achieved 99% classification accuracy across five specimen groups (500 samples total) with fixed defect positions, using an 80% training set (400 samples). Defect localization attained 100% accuracy under identical data partitioning, where CNN-extracted time–frequency features from vibration signals enabled precise determination of debonding positions relative to anchorage surfaces. Multi-defect quantification with uniformly distributed total lengths similarly achieved perfect classification (100% accuracy), demonstrating the model’s robustness for structural health monitoring. All datasets maintained class balance through stratified randomization, with 20% of samples reserved for validation.

## 6. Conclusions

This study addresses the critical issues of debonding and slippage in GFRP rock bolts by developing detection methods for interfacial defects through combined numerical simulations and experimental tests. COMSOL-based modeling and laboratory investigations are employed to systematically examine how defect length, position, and multiplicity influence signal characteristics, including amplitude, frequency, and energy distribution.

(1) Numerical simulations reveal distinct debonding patterns: Increasing defect length raises signal amplitude by 10.96% (from 0.727682 V to 0.807453 V), reduces peak frequency to 84,486 Hz, and intensifies energy concentration (peak energy: 1.3186). Variations in defect position maintain stable amplitude (±0.15%) while increasing peak frequency (from 80,987 Hz to 84,986 Hz) and energy. More defects reduce amplitude by 16.68%, elevate peak frequency to 87,320 Hz, and redistribute energy.
(2) Experimental tests confirm these trends: Longer defects enhance amplitude by 54.9%, increase energy concentration by 13.1%, but reduce peak frequency to 7083 Hz while worsening waveform distortion. Changes in defect location preserve amplitude stability (±1.19%) but elevate peak frequency and energy. Multiple defects decrease amplitude by 3.0%, increase peak frequency to 7803 kHz, and maintain stable energy despite intensified aliasing.
(3) A CNN-SVM model was developed to evaluate debonding conditions, using time–frequency representations of experimental signals as input. The model achieved exceptional accuracy rates of 99%, 100%, and 100% across three test scenarios, all surpassing the 95% reliability threshold. These results demonstrate the model’s robust capability for precise defect assessment in GFRP anchorage systems.

This study examined debonding length, location, and defect number as isolated variables. The coupling effects of these factors as well as the influence of defect type on signal peaks remain to be investigated in future work.

## Figures and Tables

**Table 1:** Numerical simulation test model material parameters.

Material	E/GPa	G/GPa	*ν*	*ρ*/(kg·m^−3^)
GFRP Bolts	(41, 25, 25)	(29.95, 29.95, 29.95)	(0.22, 0.22, 0.22)	2500
Rock mass	40	—	0.25	2700

**Table 2:** Structural parameters and performance metrics of CNN-SVM models.

Layers	Kemel Size	Number of Kernels	Stride
Convolution_1	(3, 3)	10	(1, 1)
Max_pooling_1	(2, 2)	10	(2, 2)
Convolution_2	(5, 5)	24	(1, 1)
Max_pooling_2	(2, 1)	24	(2, 2)
Dense_1		64	(1, 1)
Dense_2		32	(1, 1)
Dense_3		numClasses	(1, 1)

## Data Availability

Data will be made available on request.
